# A Digital Clinical Records Versus Paper Records in Dental Practice: A Comparative Study

**DOI:** 10.1155/bmri/5527391

**Published:** 2025-12-28

**Authors:** Antonio Scarano, Francesco Inchingolo, Gianna Dipalma, Filiberto Mastrangelo

**Affiliations:** ^1^ Department of Innovative Technology in Medicine and Dentistry, University of Chieti-Pescara, Chieti, Abruzzo, Italy, unich.it; ^2^ Department of Interdisciplinary Medicine, University of Bari “Aldo Moro”, Bari, Puglia, Italy, uniba.it; ^3^ Department of Clinical and Experimental Medicine, University of Foggia, Foggia, Puglia, Italy, unifg.it

**Keywords:** eHealth, electronic health records, electronic medical records, electronic patient records, healthcare, review, software for dentistry

## Abstract

**Background:**

Efficient analysis of patient histories is essential for dental treatment planning. Electronic health records (EHRs) facilitate the immediate identification of risk factors, such as medication allergies and sensitivities to dental materials, thereby reducing the risk of medical errors.

**Objective:**

This study is aimed at comparing the effectiveness of an EHR interface with traditional paper medical records (PMRs) in evaluating the medical histories of dental patients.

**Methods:**

Two hundred patient records were randomly assigned to either the PMR or EHR group. Twenty dental students reviewed the records and reported the planned dental procedure for each patient. Recording and reporting times, as well as the number of oversights, were measured for both groups.

**Results:**

Students using EHRs identified critical systemic conditions more rapidly and with greater accuracy, with no oversights detected. In contrast, students using PMRs required more time and were more likely to overlook important health information. The mean number of oversights in the PMR group was significantly higher (9.3 ± 0.46, *p* ≤ 0.01).

**Conclusion:**

The use of EHRs in dental practice significantly improves the identification of critical systemic conditions, enhances patient safety, and increases clinical efficiency. Despite the benefits, the high costs of software acquisition, staff training, and technical support may pose challenges to widespread adoption.

## 1. Introduction

Over the past two decades, electronic health records (EHRs) have revolutionized healthcare organizations driven by the rapid advancement of information and communication technology (ICT) worldwide [1]. The EHR provides healthcare providers with immediate access to patient histories, including treatments, medications, and blood chemistry tests. Real‐time access enhances the quality of care by enabling more accurate treatment planning. The digitization of health records has streamlined administrative processes, reducing the time and effort required to search for patient information. This efficiency allows healthcare providers to focus on patient care [2]. Management software for dentistry encompasses a wide range of applications designed to enhance the diagnostic accuracy and treatment plans. It has been suggested that the use of EHR in dental practice can improve both the quality of care and economic outcomes. The use of EHR systems in dental practice has demonstrated potential economic benefits. One study documented that EHR improved economic outcomes when running a medical clinic [3].

The use of management software in dental practice can bring numerous advantages, improving operational efficiency and the quality of service offered to patients. Management software allows for a more efficient organization and management of appointments, reducing the risk of overlaps and missed appointments. Patients can receive automatic reminders via mail or messages, improving punctuality and reducing no‐shows. It enables a secure and easily accessible digital archive of patient records including previous treatments, x‐rays, and medical notes. This facilitates the dentist′s organization and improves the continuity of care. It automates the billing and payment management processes, reduces errors, simplifies accounting by reducing unpaid services, and helps monitor the practice′s income and expenses. The EHR helps keep track of dental materials, ensuring that the practice is always well supplied, and reduces the risk of disruptions due to a lack of supplies. It monitors the use of medical devices that Italian law requires to be tracked by production lot codes in accordance with the Medical Device Regulations 2017/745 [4]. This has made work in dental clinics that will have to provide information about the medical devices used on the patient very complicated. Work that has been streamlined by the software both at the medical device registration stage and at the research or report stage is required by local health authorities and increases patient safety. The goal is to report the incidence of adverse events; in fact, reported safety alerts and field safety alerts have increased significantly over the past two decades. Management software improves communication with patients by sending emails, text messages, or push notifications for updates, reminders, and important information.

The system ensures compliance with privacy and data security regulations to protect patients’ sensitive information. It also provides analysis and reporting tools that help monitor dental practice performance, identify areas for improvement, and make informed decisions based on data. However, the high costs associated with software purchases, training, and staff time are significant obstacles in many medical fields. However, this is not an obstacle in dentistry because dentists are accustomed to handling software for implant surgical programming, CAD/CAM, etc. [5–7]. Despite these costs, the adoption of EHR in dental practices has been higher than that in medical offices, suggesting recognition of their potential economic benefits [8]. The objective of the present study was to evaluate the impact of using new dedicated software to reduce the operative time, acquire medical information, and avoid complications.

## 2. Materials and Methods

In this study, we developed a software designed to quickly identify patients with severe systemic conditions, such as allergies, use of antiresorptive medications, and uncontrolled diabetes. Each time the software is opened, it highlights patients with outstanding issues on the main screen. When a patient′s clinical record is accessed, any systemic condition requiring maximum caution by the dentist is prominently displayed. The interface was designed to be intuitive, allowing dental professionals to easily navigate and retrieve patient information. In this study, 200 clinical records were digitized using Dental Care software, resulting in a total of 200 paper records and 200 records in digital format. Twenty volunteer dental students were invited to open clinical records to report the treatment plan to the dentist. The first question posed to students was to determine the procedure to be performed on the current day. The 200 patients were randomized, and 20 volunteer dental students were asked to open the clinical records of the patients selected using the randomization system available online (https://ctrandomization.cancer.gov/tool/) allocating into two different groups: paper medical record (PMR) and EHR.

The software immediately flags patients with critical systemic conditions, ensuring that dentists are aware of any potential complications before beginning the treatment. The second question posed to the students was about recording a medical device using both management software and traditional PMRs. Afterwards, they were asked to compile a report on the implants placed in 200 patients.

### 2.1. Statistical Analysis

The data management and analysis have been conducted using a specially designed form with GraphPad 9 (Prism, San Diego, California, United States). The descriptive statistics considered cumulative numerosity, means, standard deviations, and 95% confidence intervals.

The Kolmogorov–Smirnov normality test has been applied, and the variables have been tested by conducting the nonparametric Mann–Whitney model. The level of significance has been considered for *p* value < 0.05.

## 3. Results

Students were asked to review papers and digital medical records to identify the treatment needed for each patient. Digital medical records provided immediate access to health information such as systemic conditions and medication use, and serious conditions were prominently highlighted when the digital medical record was opened. Each student then paid close attention to the highlighted messages about the patients′ critical conditions, ensuring that they were not overlooked before treatment. During the study, all students observed the highlighted message regarding the pathology that required maximum attention before dental treatment when opening the electronic clinical record and focused on the specific condition affecting the patient. None of the students paid full attention to the highlighted pathology message. When students opened medical records of healthy patients, some occasionally focused on reporting unpaid services. When using EHR, students quickly identified the systemic conditions, demonstrating the effectiveness of the software in highlighting critical health issues with no detected oversights. In contrast, when reviewing the paper records, students had to manually search find relevant health information. This process was more time‐consuming and prone to errors, as medical critical conditions could be easily overlooked. The oversight mean of the PMR group was 9.3 ± 0.46 (95% CI: 9.21; 9.39).

When students were asked to open paper clinical records, there were significant oversights:

1. Twenty‐four cases of antiresorptive medication use went unnoticed.
2. Six cases of patients on anticoagulant therapy went unnoticed.
3. One case of penicillin allergies was overlooked.

Students took significantly less time to identify critical conditions using electronic records, as the software immediately highlighted these issues.

Identifying pathologies in paper records took approximately 2 min of reading per record, highlighting the inefficiency and potential for error in manual processes.

While for the second question posed to students, for patients with digital medical records, report generation was almost instantaneous, while device registration took a total of 40 min for 100 patients, with a mean of6.6 ± 0.14(5.97; 6.03) (*p* ≤ 0.01) for the EHR group.

**Figure 1:**
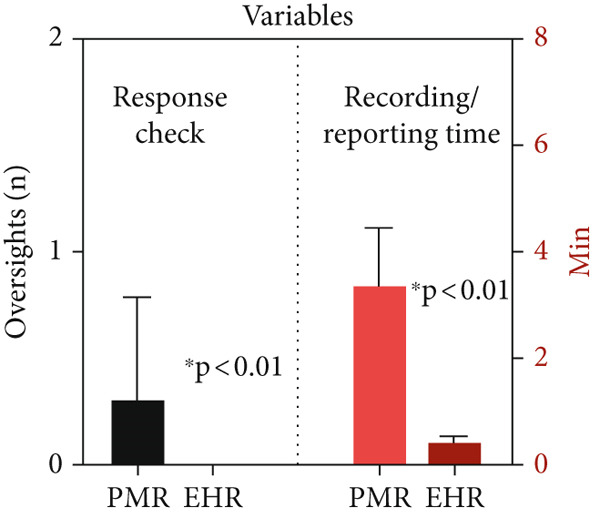
Summary chart of the study variables based on oversights measured in the response check and the time required for recording and reporting the medical history (PMR, paper medical record; EHR, electronic medical record).

In contrast, for those with PMRs, students took 112 min for the initial registration and another 213 min to complete the full report (*p* ≤ 0.01). This comparison highlights the significant time‐saving advantages of using EHR over traditional paper methods, especially in the management of patients with major medical conditions and tracking of medical devices (Figure 1, Table 1). Students who used EHR identified critical medical conditions more quickly and with fewer errors compared to those who used PMR, demonstrating the software′s effectiveness in highlighting health issues. The time taken to record and report information was significantly lower with EHR.

**Table 1:** Summary table of the study variables based on oversights measured in the response check and the time required for recording and reporting the medical history (PMR, paper medical record; EHR, electronic medical record).

	**Oversights**		**Recording/reporting time**	
	**PMR**	**EHR**	**PMR**	**EHR**
Mean	0.31	(‐)	6.6	0.78
Std. deviation	0.46	(‐)	33	3.9
Std. error of mean	0.046	(‐)	3.3	0.39
Lower 95% CI of mean	0.22	(‐)	0.13	0.016
Upper 95% CI of mean	0.40	(‐)	13	1.6

## 4. Discussion

The results of the present study show that EHR significantly improved the efficiency and accuracy of identifying necessary treatments, as all 100 patients with systemic conditions were correctly identified using the digital format. However, when using paper records, there were notable oversights, including missed cases of antiresorptive medication use, anticoagulant therapy, and penicillin allergy. The present study demonstrated that the use of EHR in dental practice enhances the ability to quickly and accurately identify critical health conditions, leading to better treatment planning and improved patient outcomes. The evidence of the present investigation was that the EHR revealed a statistically significant reduction of the recording/reporting time with a consequent improvement of the cost‐effectiveness of the process. At the same time, the EHR process seems to drastically avoid the oversight risk shown by the PMR analysis. EHR not only saves time but also reduces the risk of errors, improving the efficiency of clinical workflows. A recent study demonstrates a significant reduction in oversights (errors/oversights) with the use of EHRs compared to PMRs [9]. The user interface and ease of use of the management software are crucial factors in choosing the correct product for a dental clinic. A well‐designed interface allows simple and intuitive navigation. By highlighting critical health conditions, the software helps to prevent potential complications during dental procedures. A well‐designed interface and easy‐to‐use software allow for simple and intuitive navigation, simplifying daily operations such as data entry, appointment scheduling, and administrative management. This means that the staff can quickly access the necessary features, reducing the time spent searching for information and increasing the time dedicated to patients. When the software is intuitive, staff are more likely to adopt and use it effectively with reduced errors. This translates to lower training costs and quicker return on investment. This facilitates the integration of software into a clinic′s daily routine [10].

In summary, a user‐friendly interface and ease of use are essential to ensure that management software improves operational efficiency, staff satisfaction, and ultimately the quality of service offered to patients [11]. Financial barriers and the need for technical support are significant concerns among dental professionals [12]. EHR offers numerous advantages in the dental sector, improving both the quality of care and the overall management of dental practice.

This is particularly useful for managing patients with chronic conditions or allergies [13, 14].

DRs ensure greater data security compared to paper records owing to encryption systems and regular backups, which reduce the risk of loss or theft of sensitive information.

They allow tracking of all operations performed, improving transparency and professional accountability. In the case of disputes, it is easy to trace back to the clinical decisions made.

The information contained in EHR can be easily shared with other specialists, thereby improving interdisciplinary collaboration and continuity of care.

These advantages make electronic dental records an indispensable tool for modern dental practice, contributing to improved efficiency and quality of care offered to patients [15].

Management software for dentistry plays a crucial role in enhancing educational outcomes, improving diagnostic accuracy, streamlining prosthetic designs, and facilitating patient communication. Evidence demonstrates the effectiveness of various software applications in dental education, CAD/CAM prosthetics, orthodontic and implantology planning, caries detection, and patient compliance.

The integration of EHR into dental education allows for the collection of comprehensive patient data, which can be used for research and evaluation of the impact of clinical care on oral health outcomes [16]. Additionally, the use of EHR in dental clinics and schools can help standardize diagnostic codes and clinical outcome measures, making them more useful in clinical research [5]. EPRs offer the possibility of using big data to promote public oral health [17]. EHR plays a key role in clinical decision support systems by providing accurate and structured data. The use of electronic records can improve clinical outcomes [5, 18]. In addition, EHRs can improve the early diagnosis of Sjögren′s disease [19] and prevent osteonecrosis by intercepting patients who have taken antiresorptive drugs, thereby demonstrating the potential of EHR to improve predictive models and early intervention strategies. The EHR is also a medicolegal document that describes a clinical procedure and provides a detailed outline of the treatment plan to which the patient has been subjected, as well as providing all the number codes required by Medical Device Regulations 2017/7 [4, 20]. These are very important aspects, especially when the dental profession is burdened with numerous bureaucratic tasks, such as specific consent, medical device traceability, and the archiving of radiographs and photographs. However, the design of EHR systems, particularly the implementation of alert systems, plays a crucial role in reducing errors in clinical practice. These alert systems provide real‐time notifications and reminders to healthcare providers, ensuring that critical information is not overlooked. Human–computer interaction studies in healthcare have shown that user‐centered design principles, such as intuitive interfaces and effective feedback mechanisms, significantly improve the usability and reliability of medical software [21].

## 5. Conclusion

The adoption of EHR in dental practice significantly improves administrative efficiency by reducing registration and reporting times compared to PMR. It enhances patient safety by minimizing diagnostic oversights through automated highlighting of critical conditions. However, successful implementation requires addressing challenges such as training needs and potential data entry errors. While EHR systems improve workflow efficiency, paper records may still offer advantages in completeness for specific types of documentation.

## Disclosure

The literature review was conducted using Causaly AI, and data analysis was performed with Rayyan AI. All data were independently verified by the authors.

## Conflicts of Interest

The authors declare no conflicts of interest.

## Funding

No funding was received for this manuscript.

## Supporting information


**Supporting Information** Additional supporting information can be found online in the Supporting Information section.

## Data Availability

The data that support the findings of this study are available from the corresponding author upon reasonable request.
