# Supplementary material for: A Digital Clinical Records Versus Paper Records in Dental Practice: A Comparative Study
Source: Biomed Res Int. 2025 Dec 28;2025:5527391. doi: 10.1155/bmri/5527391 (PMC12745653; doi:10.1155/bmri/5527391)
Supplement: Supplementary file 1 — Supporting Information Additional supporting information can be found online in the Supporting Information section. [file BMRI-2025-5527391-s001.docx]

*December 1, 2025*

Dear Editor,

Dear Editor,

Thank you very much for reviewing our manuscript. According to the guidelines for the manuscript processing, please find attached the revised version of the paper with the integrations highlighted in yellow and those removed in red.

Hoping that the manuscript could be acceptable for publication,

I remain sincerely yours.

1. The last word file contains track changes, comments , highlights inside the manuscript, so kindly remove the track changes, comments, highlights and provide us with a clean version of the manuscript under the main manuscript section.

**Answer:** All track changes, comments , highlights was removed.

2. The affiliation in the manuscript should contain the following (1) the name of the department, (2) the name of the institution, and (3) the city and state, if applicable. Please update the same in the system and final Manuscript (in DOC format) under the manuscript section.. Ensure the same affiliations are provided in the system and the revised version.

**Answer:** All affiliations have been updated and are now correct.

3. Please kindly remove the one set of references from the manuscript.

**Answer:** All duplicate references have been removed from the manuscript

1. *Inconsistent Terminology: The terms "electronic medical records (EPR)" and "electronic health records (EHR)" are used interchangeably and inconsistently. The standard term is "electronic health records (EHR)" or "electronic dental records (EDR)" in dentistry.*

Answer: The suggested correction has been successfully integrated into the main text for improved clarity.

*2) Grammar and Syntax Errors:*

Answer: The suggested correction has been accepted.

3) Repetition: The conclusion repeats the phrase "avoiding the oversights that has been detected with paper MR avoiding the oversights detected with paper medical records."

Answer: the suggest change has been made.

4) Ambiguity in Results: The confidence interval [95% CI: 0.22; 0.4] does not match the mean value (9.3±0.46), which creates confusion.

Answer: the data has been corrected.

5) Unclear Group Labels: The group names (EPR vs PMR) are not clearly defined or used consistently.

Answer: the terminology has been uniformed.

***6)*** Unclear Statistical Reporting: The statistical significance and confidence intervals are not clearly or accurately reported.

Answer: The statistical reporting has been checked and corrected.

7) . Awkward Phrasing: Sentences such as "students of electronic medical records (EPR) group quickly identified the systemic conditions, demonstrating the effectiveness of the software in highlighting critical health issues with no detected oversights" are awkward and could be made clearer.

Answer: the suggest change has been made.

8) Conclusion Structure: The conclusion is too long and contains run-on sentences, making it difficult to follow.

Answer: the conclusion has been shorted

9) The abstract has been replaced with the version suggested by the reviewers.

Prof. Antonio Scarano

Department of Innovative Technologies in Medicine and Dentistry

University of Chieti-Pescara, Via dei Vestini 31, 66100 Chieti, Italy.

Telephone: +39-08713554100 -08713554099

Email: [ascarano@unich.it](mailto:ascarano@unich.it).
